# Diethyl 2,2′-[(5-dimethyl­amino-1-naphth­yl)sulfonyl­imino]diacetate

**DOI:** 10.1107/S1600536809041476

**Published:** 2009-10-17

**Authors:** Yong Zhang, Yuan Qu, Ting Liu

**Affiliations:** a School of Chemical and Materials Engineering, Huangshi Institute of Technology, Huangshi 435003, People’s Republic of China

## Abstract

In the title compound, C_20_H_26_N_2_O_6_S, the N atom of the dimethyl­amino group is displaced by 0.113 (2) Å from the plane of the naphthalene ring system. The two eth­oxy groups adopt zigzag conformations. In the crystal structure, weak inter­molecular C—H⋯O hydrogen bonds link the mol­ecules, forming a three-dimensional network. Both ethyl groups are disordered over two sites with the ratios of refined occupancies being 0.857 (16):0.143 (16) and 0.517 (14):0.483 (14).

## Related literature

For applications of ligands containing the 5-(dimethyl­amino) naphthalene-1-sulfonyl (dans­yl) group, see: Corradini *et al.* (1997 ▶); Christoforou *et al.* (2006 ▶); Zhang *et al.* (2009 ▶).
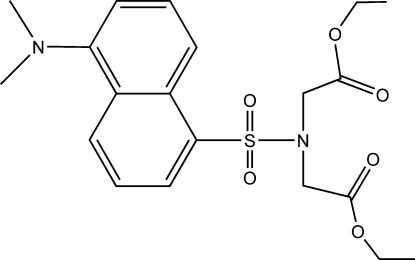


## Experimental

### 

#### Crystal data

C_20_H_26_N_2_O_6_S
*M* *_r_* = 422.49
Monoclinic, 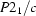

*a* = 13.1266 (9) Å
*b* = 8.4592 (5) Å
*c* = 19.3206 (12) Å
β = 93.530 (1)°
*V* = 2141.3 (2) Å^3^
*Z* = 4
Mo *K*α radiation
μ = 0.19 mm^−1^
*T* = 298 K
0.20 × 0.20 × 0.20 mm

#### Data collection

Bruker SMART CCD diffractometer
Absorption correction: multi-scan (*SADABS*; Sheldrick, 1996 ▶) *T*_min_ = 0.970, *T*_max_ = 0.981
16359 measured reflections
4201 independent reflections
3705 reflections with *I* > 2σ(*I*)
*R*_int_ = 0.040

#### Refinement

*R*[*F* ^2^ > 2σ(*F* ^2^)] = 0.053
*wR*(*F* ^2^) = 0.145
*S* = 1.05
4201 reflections
306 parameters
12 restraints
H-atom parameters constrained
Δρ_max_ = 0.35 e Å^−3^
Δρ_min_ = −0.27 e Å^−3^

### 

Data collection: *SMART* (Bruker, 2007 ▶); cell refinement: *SAINT-Plus* (Bruker, 2007 ▶); data reduction: *SAINT-Plus*; program(s) used to solve structure: *SHELXS97* (Sheldrick, 2008 ▶); program(s) used to refine structure: *SHELXL97* (Sheldrick, 2008 ▶); molecular graphics: *PLATON* (Spek, 2009 ▶); software used to prepare material for publication: *SHELXTL* (Sheldrick, 2008 ▶).

## Supplementary Material

Crystal structure: contains datablocks global, I. DOI: 10.1107/S1600536809041476/lh2912sup1.cif

Structure factors: contains datablocks I. DOI: 10.1107/S1600536809041476/lh2912Isup2.hkl

Additional supplementary materials:  crystallographic information; 3D view; checkCIF report

## Figures and Tables

**Table 1:** Hydrogen-bond geometry (Å, °)

*D*—H⋯*A*	*D*—H	H⋯*A*	*D*⋯*A*	*D*—H⋯*A*
C4—H4⋯O1	0.93	2.36	3.006 (3)	126
C13—H13*A*⋯O2^i^	0.97	2.36	3.272 (3)	157

Symmetry code: (i) 
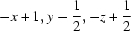
.

**Fig. 1.**
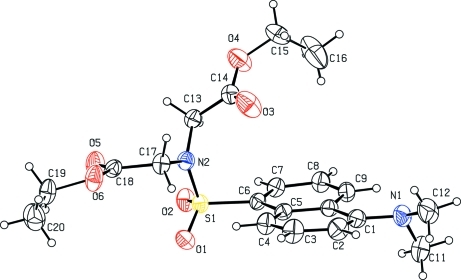
The molecular structure of (I), with displacement ellipsoids drawn at the 50% probability level. The disorder is not shown.

**Fig. 2.**
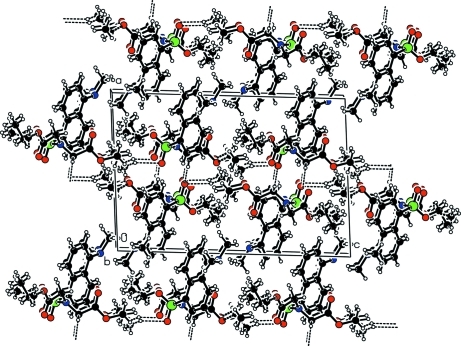
Part of the crystal structure of (I) showing weak hydrogen bonds as dashed lines.

## supplementary crystallographic information

### Comment

Dansyl (5-(dimethylamino)naphthalene-1-sulfonyl) derivatives are of considerable interest because of their good fluorescent properties. Many fluorescent ligands bearing dansyl group have been reported in recent years (e.g. Corradini *et al.*, 1997; Christoforou *et al.*, 2006; Zhang *et al.*, 2009). We are interested in preparing fluorescent ligands that are expected to bind to hydrophobic sites in proteins or membranes. With this in mind, the title compound, (I), was prepared and we report herein the crystal stucture.

In the molecular structure (Fig. 1), the N atom of the dimethylamino group is displaced by 0.113 (2) Å from the plane of the naphthalene ring system. The two ethoxycarbonyl groups adopt coiled conformations with C14—O4—C15—C16 and C18—O6—C119—C20 torsion angles of 90.1 (10)° and 159.6 (9)°, respectively. All bond lengths and bond angles are as expected. In the crystal structure (Fig.2) weak intermolecular C—H···O hydrogen bonds lead to the formation of a three-dimension network.

### Experimental

Diethyl iminodiacetate(0.38 g, 2 mmol) was added to a stirred solution of dansyl chloride(0.27 g, 1 mmol) in dry acetonitrile(40 ml). The reaction mixture was allowed to stir for 12 hr at 353 K. The progress of the reaction was monitored by TLC, untill the completion of reaction. The solvent was evaporated and the residue was purified by column chromatography (hexane-ethyl acetate,1:5 *v*/*v*) to afford the title compound as a yellow solid. Single crystals suitable for X-ray diffraction were prepared by slow evaporation of a solution of the title compound in ethyl acetate at room temperature.

### Refinement

Both the C15/C16 and C18/C19 ethyl groups are disordered over two positions with the final refined occupancies of 0.857 (16):0.143 (16) and 0.517 (14):0.483 (14) for the major and minor components, respectively. In the refinement, commands 'DFIX' and 'SADI' were used (Sheldrick, 2008). Some B-level alert using *PLATON* were resulted from the disorder.

All H atoms were placed in idealized positions [C–H=0.96 Å (methyl), 0.97Å (methylene) and 0.93 Å (aromatic)] and included in the refinement in the riding-model approximation, with *U*_iso_(H)= 1.5*U*_eq_(methyl C) and 1.2*U*_eq_(methylene and aromatic C).

### Crystal data

**Table:**

C_20_H_26_N_2_O_6_S	*F*(000) = 896
*M**_r_* = 422.49	*D*_x_ = 1.311 Mg m^−^^3^
Monoclinic, *P*2_1_/*c*	Mo *K*α radiation, λ = 0.71073 Å
Hall symbol: -P 2ybc	Cell parameters from 7099 reflections
*a* = 13.1266 (9) Å	θ = 2.4–27.8°
*b* = 8.4592 (5) Å	µ = 0.19 mm^−^^1^
*c* = 19.3206 (12) Å	*T* = 298 K
β = 93.530 (1)°	Block, yellow
*V* = 2141.3 (2) Å^3^	0.20 × 0.20 × 0.20 mm
*Z* = 4	

### Data collection

**Table:**

Bruker SMART CCD diffractometer	4201 independent reflections
Radiation source: fine-focus sealed tube	3705 reflections with *I* > 2σ(*I*)
graphite	*R*_int_ = 0.040
φ and ω scans	θ_max_ = 26.0°, θ_min_ = 2.5°
Absorption correction: multi-scan (*SADABS*; Sheldrick, 1996)	*h* = −15→16
*T*_min_ = 0.970, *T*_max_ = 0.981	*k* = −10→10
16359 measured reflections	*l* = −23→23

### Refinement

**Table:**

Refinement on *F*^2^	Primary atom site location: structure-invariant direct methods
Least-squares matrix: full	Secondary atom site location: difference Fourier map
*R*[*F*^2^ > 2σ(*F*^2^)] = 0.053	Hydrogen site location: inferred from neighbouring sites
*wR*(*F*^2^) = 0.145	H-atom parameters constrained
*S* = 1.05	*w* = 1/[σ^2^(*F*_o_^2^) + (0.0753*P*)^2^ + 0.7093*P*] where *P* = (*F*_o_^2^ + 2*F*_c_^2^)/3
4201 reflections	(Δ/σ)_max_ < 0.001
306 parameters	Δρ_max_ = 0.35 e Å^−^^3^
12 restraints	Δρ_min_ = −0.27 e Å^−^^3^

### Special details

**Table:**

Geometry. All e.s.d.'s (except the e.s.d. in the dihedral angle between two l.s. planes) are estimated using the full covariance matrix. The cell e.s.d.'s are taken into account individually in the estimation of e.s.d.'s in distances, angles and torsion angles; correlations between e.s.d.'s in cell parameters are only used when they are defined by crystal symmetry. An approximate (isotropic) treatment of cell e.s.d.'s is used for estimating e.s.d.'s involving l.s. planes.
Refinement. Refinement of *F*^2^ against ALL reflections. The weighted *R*-factor *wR* and goodness of fit *S* are based on *F*^2^, conventional *R*-factors *R* are based on *F*, with *F* set to zero for negative *F*^2^. The threshold expression of *F*^2^ > σ(*F*^2^) is used only for calculating *R*-factors(gt) *etc*. and is not relevant to the choice of reflections for refinement. *R*-factors based on *F*^2^ are statistically about twice as large as those based on *F*, and *R*- factors based on ALL data will be even larger.

### Fractional atomic coordinates and isotropic or equivalent isotropic displacement parameters (Å^2^)

**Table:**

	*x*	*y*	*z*	*U*_iso_*/*U*_eq_	Occ. (<1)
C1	0.95264 (17)	0.5912 (3)	0.37909 (11)	0.0578 (5)	
C2	1.02352 (18)	0.4871 (3)	0.35796 (14)	0.0715 (7)	
H2	1.0911	0.4969	0.3750	0.086*	
C3	0.99669 (18)	0.3659 (3)	0.31122 (15)	0.0737 (7)	
H3	1.0466	0.2959	0.2981	0.088*	
C4	0.89929 (16)	0.3482 (3)	0.28453 (13)	0.0622 (6)	
H4	0.8836	0.2689	0.2523	0.075*	
C5	0.82149 (15)	0.4499 (2)	0.30558 (10)	0.0466 (4)	
C6	0.71621 (15)	0.4373 (2)	0.28232 (10)	0.0446 (4)	
C7	0.64297 (16)	0.5326 (2)	0.30780 (11)	0.0522 (5)	
H7	0.5752	0.5227	0.2912	0.063*	
C8	0.67007 (17)	0.6443 (3)	0.35867 (12)	0.0588 (5)	
H8	0.6200	0.7065	0.3771	0.071*	
C9	0.76932 (17)	0.6628 (3)	0.38138 (12)	0.0575 (5)	
H9	0.7861	0.7382	0.4153	0.069*	
C10	0.84776 (15)	0.5708 (2)	0.35492 (10)	0.0490 (5)	
C11	0.9684 (3)	0.8725 (4)	0.39713 (18)	0.0941 (10)	
H11A	1.0269	0.8936	0.3712	0.141*	
H11B	0.9651	0.9487	0.4337	0.141*	
H11C	0.9076	0.8794	0.3669	0.141*	
C12	1.0732 (2)	0.6972 (5)	0.46859 (16)	0.0957 (10)	
H12A	1.0791	0.5906	0.4854	0.144*	
H12B	1.0742	0.7688	0.5072	0.144*	
H12C	1.1293	0.7203	0.4406	0.144*	
C13	0.59995 (17)	0.1130 (3)	0.32052 (11)	0.0548 (5)	
H13A	0.5668	0.0108	0.3159	0.066*	
H13B	0.5478	0.1937	0.3136	0.066*	
C14	0.64919 (19)	0.1283 (3)	0.39272 (12)	0.0588 (5)	
C15	0.6061 (8)	0.1733 (7)	0.5102 (2)	0.0874 (19)	0.857 (16)
H15A	0.6740	0.1337	0.5230	0.105*	0.857 (16)
H15B	0.5583	0.1241	0.5399	0.105*	0.857 (16)
C16	0.6032 (10)	0.3472 (8)	0.5176 (3)	0.149 (4)	0.857 (16)
H16A	0.6565	0.3935	0.4925	0.223*	0.857 (16)
H16B	0.6128	0.3748	0.5658	0.223*	0.857 (16)
H16C	0.5383	0.3861	0.4994	0.223*	0.857 (16)
C17	0.73411 (17)	−0.0060 (3)	0.24933 (12)	0.0552 (5)	
H17A	0.7484	−0.0699	0.2905	0.066*	
H17B	0.7988	0.0315	0.2339	0.066*	
C18	0.68302 (16)	−0.1075 (2)	0.19336 (11)	0.0520 (5)	
C19	0.7087 (7)	−0.2942 (12)	0.1072 (5)	0.073 (3)	0.517 (14)
H19A	0.6722	−0.2204	0.0762	0.088*	0.517 (14)
H19B	0.6624	−0.3784	0.1185	0.088*	0.517 (14)
C20	0.8001 (6)	−0.3609 (15)	0.0734 (5)	0.100 (3)	0.517 (14)
H20A	0.8432	−0.2759	0.0600	0.150*	0.517 (14)
H20B	0.7776	−0.4207	0.0331	0.150*	0.517 (14)
H20C	0.8378	−0.4285	0.1057	0.150*	0.517 (14)
C15'	0.626 (2)	0.223 (5)	0.4978 (12)	0.069 (9)	0.143 (16)
H15C	0.6659	0.3117	0.4825	0.083*	0.143 (16)
H15D	0.6711	0.1531	0.5253	0.083*	0.143 (16)
C16'	0.539 (3)	0.281 (6)	0.5403 (16)	0.122 (14)	0.143 (16)
H16D	0.5088	0.3735	0.5189	0.182*	0.143 (16)
H16E	0.5652	0.3060	0.5864	0.182*	0.143 (16)
H16F	0.4883	0.1993	0.5421	0.182*	0.143 (16)
C19'	0.7221 (11)	−0.3420 (9)	0.1268 (4)	0.069 (3)	0.483 (14)
H19C	0.6507	−0.3697	0.1290	0.083*	0.483 (14)
H19D	0.7638	−0.4342	0.1382	0.083*	0.483 (14)
C20'	0.7438 (12)	−0.2768 (12)	0.0564 (4)	0.101 (4)	0.483 (14)
H20D	0.7039	−0.1831	0.0474	0.152*	0.483 (14)
H20E	0.7263	−0.3544	0.0215	0.152*	0.483 (14)
H20F	0.8150	−0.2515	0.0556	0.152*	0.483 (14)
N1	0.97658 (16)	0.7153 (3)	0.42652 (11)	0.0706 (6)	
N2	0.67264 (13)	0.1287 (2)	0.26716 (9)	0.0524 (4)	
O1	0.74296 (13)	0.2660 (2)	0.16995 (8)	0.0631 (4)	
O2	0.56924 (12)	0.3303 (2)	0.20057 (8)	0.0635 (4)	
O3	0.73809 (15)	0.1308 (3)	0.40732 (10)	0.0988 (7)	
O4	0.57813 (15)	0.1376 (3)	0.43757 (9)	0.0934 (7)	
O5	0.59653 (12)	−0.0973 (2)	0.17180 (10)	0.0751 (5)	
O6	0.74949 (13)	−0.2111 (2)	0.17216 (11)	0.0783 (6)	
S1	0.67202 (4)	0.28980 (6)	0.22222 (3)	0.04759 (18)	

### Atomic displacement parameters (Å^2^)

**Table:**

	*U*^11^	*U*^22^	*U*^33^	*U*^12^	*U*^13^	*U*^23^
C1	0.0469 (12)	0.0728 (14)	0.0531 (12)	−0.0099 (10)	−0.0019 (9)	0.0004 (10)
C2	0.0377 (12)	0.0952 (19)	0.0807 (17)	−0.0057 (12)	−0.0047 (11)	−0.0049 (14)
C3	0.0409 (12)	0.0834 (18)	0.0969 (19)	0.0088 (12)	0.0039 (12)	−0.0136 (15)
C4	0.0439 (12)	0.0645 (13)	0.0783 (15)	0.0032 (10)	0.0028 (10)	−0.0126 (12)
C5	0.0390 (10)	0.0492 (10)	0.0516 (11)	−0.0017 (8)	0.0024 (8)	0.0026 (9)
C6	0.0416 (10)	0.0439 (10)	0.0479 (10)	−0.0013 (8)	−0.0012 (8)	0.0002 (8)
C7	0.0409 (11)	0.0525 (11)	0.0625 (12)	0.0024 (9)	−0.0020 (9)	−0.0013 (10)
C8	0.0492 (12)	0.0585 (12)	0.0691 (14)	0.0070 (10)	0.0073 (10)	−0.0115 (11)
C9	0.0574 (13)	0.0585 (12)	0.0567 (12)	−0.0042 (10)	0.0027 (10)	−0.0115 (10)
C10	0.0433 (11)	0.0553 (11)	0.0481 (11)	−0.0033 (9)	−0.0003 (8)	0.0032 (9)
C11	0.094 (2)	0.086 (2)	0.100 (2)	−0.0227 (17)	−0.0109 (18)	−0.0207 (18)
C12	0.0681 (18)	0.139 (3)	0.0770 (18)	−0.0235 (18)	−0.0197 (14)	−0.0136 (18)
C13	0.0504 (12)	0.0551 (12)	0.0586 (12)	−0.0068 (9)	0.0010 (9)	−0.0018 (10)
C14	0.0591 (14)	0.0581 (13)	0.0587 (13)	0.0007 (11)	0.0002 (11)	−0.0021 (10)
C15	0.106 (4)	0.108 (4)	0.048 (2)	0.013 (3)	0.008 (2)	0.010 (2)
C16	0.252 (12)	0.115 (4)	0.075 (3)	0.068 (5)	−0.026 (4)	−0.014 (3)
C17	0.0509 (12)	0.0492 (11)	0.0642 (13)	0.0078 (9)	−0.0064 (10)	−0.0050 (9)
C18	0.0450 (12)	0.0507 (11)	0.0612 (12)	−0.0067 (9)	0.0104 (9)	−0.0046 (9)
C19	0.070 (4)	0.055 (5)	0.093 (7)	0.002 (4)	−0.003 (5)	−0.027 (5)
C20	0.091 (5)	0.108 (7)	0.103 (5)	0.003 (4)	0.019 (4)	−0.038 (5)
C15'	0.078 (16)	0.09 (2)	0.044 (12)	0.002 (13)	0.007 (11)	0.002 (13)
C16'	0.11 (3)	0.13 (3)	0.12 (2)	0.04 (2)	−0.006 (19)	0.01 (2)
C19'	0.092 (7)	0.044 (4)	0.074 (4)	−0.011 (4)	0.018 (4)	−0.005 (3)
C20'	0.153 (11)	0.085 (6)	0.067 (4)	−0.032 (6)	0.025 (5)	−0.010 (4)
N1	0.0575 (12)	0.0899 (16)	0.0630 (12)	−0.0152 (11)	−0.0086 (9)	−0.0105 (11)
N2	0.0545 (10)	0.0458 (9)	0.0570 (10)	0.0032 (8)	0.0041 (8)	−0.0042 (8)
O1	0.0666 (10)	0.0730 (10)	0.0499 (8)	0.0011 (8)	0.0046 (7)	−0.0043 (7)
O2	0.0521 (9)	0.0667 (9)	0.0688 (10)	0.0086 (7)	−0.0194 (7)	−0.0052 (8)
O3	0.0622 (12)	0.161 (2)	0.0711 (12)	0.0146 (13)	−0.0128 (9)	−0.0102 (13)
O4	0.0767 (13)	0.1434 (19)	0.0607 (11)	−0.0036 (13)	0.0095 (9)	−0.0127 (12)
O5	0.0455 (9)	0.0878 (12)	0.0910 (12)	−0.0035 (8)	−0.0051 (8)	−0.0257 (10)
O6	0.0566 (10)	0.0703 (11)	0.1090 (14)	−0.0017 (8)	0.0126 (9)	−0.0396 (10)
S1	0.0445 (3)	0.0501 (3)	0.0474 (3)	0.0023 (2)	−0.0045 (2)	−0.0020 (2)

### Geometric parameters (Å, °)

**Table:**

C1—C2	1.362 (4)	C15—H15A	0.9700
C1—N1	1.416 (3)	C15—H15B	0.9700
C1—C10	1.437 (3)	C16—H16A	0.9600
C2—C3	1.397 (4)	C16—H16B	0.9600
C2—H2	0.9300	C16—H16C	0.9600
C3—C4	1.357 (3)	C17—N2	1.450 (3)
C3—H3	0.9300	C17—C18	1.506 (3)
C4—C5	1.414 (3)	C17—H17A	0.9700
C4—H4	0.9300	C17—H17B	0.9700
C5—C10	1.426 (3)	C18—O5	1.188 (3)
C5—C6	1.431 (3)	C18—O6	1.319 (3)
C6—C7	1.369 (3)	C19—O6	1.509 (7)
C6—S1	1.777 (2)	C19—C20	1.510 (8)
C7—C8	1.394 (3)	C19—H19A	0.9700
C7—H7	0.9300	C19—H19B	0.9700
C8—C9	1.358 (3)	C20—H20A	0.9600
C8—H8	0.9300	C20—H20B	0.9600
C9—C10	1.412 (3)	C20—H20C	0.9600
C9—H9	0.9300	C15'—O4	1.477 (10)
C11—N1	1.447 (4)	C15'—C16'	1.529 (10)
C11—H11A	0.9600	C15'—H15C	0.9700
C11—H11B	0.9600	C15'—H15D	0.9700
C11—H11C	0.9600	C16'—H16D	0.9600
C12—N1	1.472 (3)	C16'—H16E	0.9600
C12—H12A	0.9600	C16'—H16F	0.9600
C12—H12B	0.9600	C19'—O6	1.444 (8)
C12—H12C	0.9600	C19'—C20'	1.511 (8)
C13—N2	1.453 (3)	C19'—H19C	0.9700
C13—C14	1.506 (3)	C19'—H19D	0.9700
C13—H13A	0.9700	C20'—H20D	0.9600
C13—H13B	0.9700	C20'—H20E	0.9600
C14—O3	1.184 (3)	C20'—H20F	0.9600
C14—O4	1.314 (3)	N2—S1	1.6160 (18)
C15—O4	1.460 (5)	O1—S1	1.4295 (17)
C15—C16	1.478 (7)	O2—S1	1.4292 (15)
			
C2—C1—N1	123.0 (2)	H15A—C15—H15B	108.6
C2—C1—C10	118.9 (2)	N2—C17—C18	112.89 (18)
N1—C1—C10	118.0 (2)	N2—C17—H17A	109.0
C1—C2—C3	121.2 (2)	C18—C17—H17A	109.0
C1—C2—H2	119.4	N2—C17—H17B	109.0
C3—C2—H2	119.4	C18—C17—H17B	109.0
C4—C3—C2	121.4 (2)	H17A—C17—H17B	107.8
C4—C3—H3	119.3	O5—C18—O6	125.1 (2)
C2—C3—H3	119.3	O5—C18—C17	125.7 (2)
C3—C4—C5	120.2 (2)	O6—C18—C17	109.14 (19)
C3—C4—H4	119.9	O6—C19—C20	106.4 (6)
C5—C4—H4	119.9	O6—C19—H19A	110.4
C4—C5—C10	118.66 (19)	C20—C19—H19A	110.4
C4—C5—C6	124.39 (19)	O6—C19—H19B	110.4
C10—C5—C6	116.92 (18)	C20—C19—H19B	110.4
C7—C6—C5	121.96 (18)	H19A—C19—H19B	108.6
C7—C6—S1	116.10 (15)	O4—C15'—C16'	106.3 (13)
C5—C6—S1	121.85 (15)	O4—C15'—H15C	110.5
C6—C7—C8	119.89 (19)	C16'—C15'—H15C	110.5
C6—C7—H7	120.1	O4—C15'—H15D	110.5
C8—C7—H7	120.1	C16'—C15'—H15D	110.5
C9—C8—C7	120.3 (2)	H15C—C15'—H15D	108.7
C9—C8—H8	119.9	C15'—C16'—H16D	109.5
C7—C8—H8	119.9	C15'—C16'—H16E	109.5
C8—C9—C10	121.7 (2)	H16D—C16'—H16E	109.5
C8—C9—H9	119.1	C15'—C16'—H16F	109.5
C10—C9—H9	119.1	H16D—C16'—H16F	109.5
C9—C10—C5	119.07 (18)	H16E—C16'—H16F	109.5
C9—C10—C1	121.5 (2)	O6—C19'—C20'	102.3 (6)
C5—C10—C1	119.39 (19)	O6—C19'—H19C	111.3
N1—C11—H11A	109.5	C20'—C19'—H19C	111.3
N1—C11—H11B	109.5	O6—C19'—H19D	111.3
H11A—C11—H11B	109.5	C20'—C19'—H19D	111.3
N1—C11—H11C	109.5	H19C—C19'—H19D	109.2
H11A—C11—H11C	109.5	C19'—C20'—H20D	109.5
H11B—C11—H11C	109.5	C19'—C20'—H20E	109.5
N1—C12—H12A	109.5	H20D—C20'—H20E	109.5
N1—C12—H12B	109.5	C19'—C20'—H20F	109.5
H12A—C12—H12B	109.5	H20D—C20'—H20F	109.5
N1—C12—H12C	109.5	H20E—C20'—H20F	109.5
H12A—C12—H12C	109.5	C1—N1—C11	114.8 (2)
H12B—C12—H12C	109.5	C1—N1—C12	115.4 (2)
N2—C13—C14	112.71 (18)	C11—N1—C12	110.7 (2)
N2—C13—H13A	109.0	C17—N2—C13	119.76 (18)
C14—C13—H13A	109.0	C17—N2—S1	121.31 (15)
N2—C13—H13B	109.0	C13—N2—S1	118.44 (14)
C14—C13—H13B	109.0	C14—O4—C15	120.0 (4)
H13A—C13—H13B	107.8	C14—O4—C15'	105.2 (9)
O3—C14—O4	124.8 (2)	C18—O6—C19'	123.6 (6)
O3—C14—C13	125.6 (2)	C18—O6—C19	111.1 (4)
O4—C14—C13	109.5 (2)	O2—S1—O1	118.12 (10)
O4—C15—C16	107.0 (4)	O2—S1—N2	109.44 (10)
O4—C15—H15A	110.3	O1—S1—N2	106.18 (9)
C16—C15—H15A	110.3	O2—S1—C6	106.70 (9)
O4—C15—H15B	110.3	O1—S1—C6	111.09 (10)
C16—C15—H15B	110.3	N2—S1—C6	104.51 (9)
			
N1—C1—C2—C3	179.8 (2)	C18—C17—N2—C13	88.7 (2)
C10—C1—C2—C3	2.7 (4)	C18—C17—N2—S1	−83.1 (2)
C1—C2—C3—C4	0.7 (4)	C14—C13—N2—C17	81.6 (2)
C2—C3—C4—C5	−2.2 (4)	C14—C13—N2—S1	−106.41 (19)
C3—C4—C5—C10	0.2 (4)	O3—C14—O4—C15	8.1 (5)
C3—C4—C5—C6	−177.7 (2)	C13—C14—O4—C15	−172.2 (3)
C4—C5—C6—C7	175.7 (2)	O3—C14—O4—C15'	26 (2)
C10—C5—C6—C7	−2.3 (3)	C13—C14—O4—C15'	−155 (2)
C4—C5—C6—S1	−0.8 (3)	C16—C15—O4—C14	90.1 (10)
C10—C5—C6—S1	−178.75 (15)	C16—C15—O4—C15'	39 (4)
C5—C6—C7—C8	−1.0 (3)	C16'—C15'—O4—C14	163 (3)
S1—C6—C7—C8	175.69 (17)	C16'—C15'—O4—C15	−62 (3)
C6—C7—C8—C9	2.2 (3)	O5—C18—O6—C19'	−9.6 (5)
C7—C8—C9—C10	−0.2 (4)	C17—C18—O6—C19'	170.0 (4)
C8—C9—C10—C5	−3.2 (3)	O5—C18—O6—C19	11.4 (6)
C8—C9—C10—C1	179.9 (2)	C17—C18—O6—C19	−169.0 (6)
C4—C5—C10—C9	−173.8 (2)	C20'—C19'—O6—C18	95.9 (12)
C6—C5—C10—C9	4.3 (3)	C20'—C19'—O6—C19	34.6 (15)
C4—C5—C10—C1	3.2 (3)	C20—C19—O6—C18	159.6 (9)
C6—C5—C10—C1	−178.75 (18)	C20—C19—O6—C19'	−72.0 (19)
C2—C1—C10—C9	172.3 (2)	C17—N2—S1—O2	126.11 (17)
N1—C1—C10—C9	−4.9 (3)	C13—N2—S1—O2	−45.80 (18)
C2—C1—C10—C5	−4.6 (3)	C17—N2—S1—O1	−2.41 (19)
N1—C1—C10—C5	178.17 (19)	C13—N2—S1—O1	−174.32 (15)
N2—C13—C14—O3	−10.0 (4)	C17—N2—S1—C6	−119.93 (17)
N2—C13—C14—O4	170.3 (2)	C13—N2—S1—C6	68.16 (17)
N2—C17—C18—O5	−10.7 (3)	C7—C6—S1—O2	14.37 (19)
N2—C17—C18—O6	169.75 (19)	C5—C6—S1—O2	−168.98 (16)
C2—C1—N1—C11	111.9 (3)	C7—C6—S1—O1	144.39 (16)
C10—C1—N1—C11	−71.0 (3)	C5—C6—S1—O1	−38.95 (19)
C2—C1—N1—C12	−18.7 (4)	C7—C6—S1—N2	−101.51 (17)
C10—C1—N1—C12	158.4 (2)	C5—C6—S1—N2	75.14 (17)

### Hydrogen-bond geometry (Å, °)

**Table:**

*D*—H···*A*	*D*—H	H···*A*	*D*···*A*	*D*—H···*A*
C4—H4···O1	0.93	2.36	3.006 (3)	126
C13—H13A···O2^i^	0.97	2.36	3.272 (3)	157

Symmetry codes: (i) −*x*+1, *y*−1/2, −*z*+1/2.

## References

[bb1] Bruker (2007). *SAINT-Plus* and *SMART* Bruker AXS Inc., Madison, Wisconsin, USA.

[bb2] Christoforou, A. M., Marzilli, P. A. & Marzilli, L. G. (2006). *Inorg. Chem.* **45**, 6771–6781. 10.1021/ic0606375 16903734

[bb3] Corradini, R., Dossena, A., Galaverna, G., Marchelli, R., Panagia, A. & Sarto, G. (1997). *J. Org. Chem.* **62**, 6283–6289.

[bb4] Sheldrick, G. M. (1996). *SADABS* University of Göttingen, Germany.

[bb5] Sheldrick, G. M. (2008). *Acta Cryst.* A**64**, 112–122. 10.1107/S0108767307043930 18156677

[bb6] Spek, A. L. (2009). *Acta Cryst.* D**65**, 148–155. 10.1107/S090744490804362X PMC2631630 19171970

[bb7] Zhang, S., Zhao, B., Su, Z., Xia, X. & Zhang, Y. (2009). *Acta Cryst.* E**65**, o1452. 10.1107/S160053680901962X PMC2969652 21583290

